# Dependencies of Agile Teams – An Analysis of the Scaled Agile Framework

**DOI:** 10.1007/978-3-030-58858-8_22

**Published:** 2020-08-18

**Authors:** Sven Theobald, Anna Schmitt

**Affiliations:** 6 IT University of Copenhagen, Copenhagen, Denmark; 7 University of British Columbia, Vancouver, BC Canada; Fraunhofer IESE, Fraunhofer-Platz 1, Kaiserslautern, Germany

**Keywords:** Agile team, Dependency, Interface, Scaled Agile Framework

## Abstract

*Context:* Agile teams are small teams with 3 to 9 members. In complex development endeavors such as systems engineering, an agile team has many dependencies, since it is not possible to incorporate all specialist skills into one team. Frameworks like the Scaled Agile Framework (SAFe) describe how agile teams operate in a larger setting. *Objective:* The aim of this study is to analyze how agile teams collaborate with their organizational environment. *Method:* We analyzed SAFe to investigate how much guidance it provides concerning the collaboration between agile teams and their environment. *Results:* The results show that many different organizational parts exist with which agile teams have to collaborate. SAFe mentions concepts like shared services, system teams, or business teams, but there is no further guidance on collaboration with the agile team. *Conclusion:* We motivate future research into guidelines for efficient collaboration of agile teams with their organizational environment.

## Introduction

Many software development teams already use agile development approaches like Scrum, eXtreme Programming or Kanban [1]. In an ideal agile environment, agile teams are small cross-functional teams that have all the competencies needed to ship product increments in regular short time intervals. They are self-empowered and can work without external dependencies. However, this ideal team setup is not always possible. For the development of large, complex products, many specialized skills are required that cannot all be part of a small agile team. Most of those roles are not required full-time, but only contribute in certain phases of the product development.

Therefore, agile software development teams have to collaborate with many different parties. In addition, teams need the support of different organizational functions like marketing, sales, or the human resources department [2]. Especially in established large companies, it is difficult to change the existing hierarchical structures, silo knowledge, and traditional mindset. For the development of safety-critical products like cars, traditional systems engineering processes are still in use and define a framework for the underlying agile, hybrid, or traditional subprojects [3]. This gap between the agile way of working of development teams and the traditional approach of the surrounding organization leads to problems regarding the collaboration between an agile team and its environment, so-called interface problems [2]. Since the environment is important in order to allow agile teams to thrive [4], efficient collaboration between agile teams and the environment on which they depend is important.

In this work, we analyze what guidance the Scaled Agile Framework (SAFe) provides regarding collaboration at the interface between an agile team and its environment. In Sect. 2, we provide further motivation for research on the collaboration between agile teams and their environment, and present the research question as well as our research approach. Section 3 presents the concepts of SAFe for synchronizing agile teams with their environment. In Sect. 4, we discuss how much guidance is provided and what guidance is lacking, and summarize the need for further research on this topic. Finally, we conclude the paper with suggestions for future work in Sect. 5.

## Motivation and Research Approach

Challenges mentioned at the workshop A-Teams in 2019 [5] were that teams have “too many dependencies to others”, a “lack of organizational support”, and “part-time resources”, which are all “interface problems” [2]. In the 2018 A-teams workshop [6], concerns like “coordination”, “organizational context supporting autonomy”, or “leadership” also address interface problems and were rated as having the highest priority.

The large-scale agile workshop [7] also identified “inter team communication” as a challenge, e.g., whether formal interfaces are needed or whether new or special roles need to be defined that intermediate between interface partners. “Agile transformation and business agility” was another challenge that deals with the alignment between business and IT, and “Scaling Agile” dealt with agile beyond software development.

Participants of the Agile Transformation workshop (A-Trans) 2019 [8] reported challenges like “hierarchical management and organizational boundaries”, “integrating non-development functions”, or “coordination challenges in multi-team environment”.

The research themes from these workshops show the need to investigate how autonomous teams collaborate with their organizational environment. Every team is dependent on organizational functions, independent of whether they are part of a scaled agile project or working as a single team. In the scaled context, additional dependencies occur. Team-level agile methods like Scrum, Kanban, or XP only discuss a few of these dependencies, namely the interface to the customer in the form of the Product Owner, as well as the Scrum Master, who shields the team from negative influences from the organization or resolves impediments caused by the environment.

In order to get a more complete picture, we wanted to investigate so-called scaling agile frameworks in terms of their support of those interfaces. We initially considered the list collected for a comparison of scaling agile frameworks [9]. We then focused on the frameworks that not only deal with scaled product development (e.g., Scrum of Scrums, LeSS, or Nexus), but also offer solutions for a complete agile organization. The expectation was that these frameworks provide support when synchronizing agile teams with their environment.

We selected the Scaled Agile Framework (SAFe), the Disciplined Agile (DA) Framework, Scrum at Scale (SaS), and the lesser known Recipes for Agile Governance (RAGE). These frameworks had the largest scope, thus we hoped to identify the most complete list of dependencies between agile teams and the rest of the organization.

For this work, we decided to focus on SAFe [10] as the most popular and most commonly used scaling framework [1]. Thus we defined the following research question:

RQ1. What guidance does SAFe provide for the collaboration between an agile team and its environment?

In order to answer this research question, the existing documentation on SAFe version 4.6 [10] was analyzed. The “Full SAFe” configuration was considered, covering all four levels (Team, Program, Large Solution, Portfolio). For each level, all concepts such as roles and practices are investigated by reading the information provided for each concept. In addition, “the foundation” of SAFe includes information about core values, the lean-agile mindset, SAFe principles, or guidelines for the implementation of SAFe. The “competencies for a lean enterprise” define five competencies regarding lean-agile leadership, team and technical agility, DevOps and release on demand, business solutions and lean systems engineering, and lean portfolio management. Finally, the “spanning palette” contains additional aspects that influence several levels, e.g., metrics, shared services, communities of practice, roadmap, or system teams.

First, a student researcher systematically went through all these parts of SAFe to identify all mentioned dependencies between an agile team and its environment and noted down all dependencies in a document. Independently, the two authors investigated SAFe regarding concepts that align the agile team with dependent organizational parts. This was not done systematically, but based on previous experiences of the authors with scaling agile frameworks in general and SAFe in particular. Afterwards, the two authors checked the dependencies found by the systematic analysis of the student researcher and had a closer look into the information that is provided by SAFe on how to handle these dependencies.

## Results

Our analysis of SAFe [10] identified several concepts regarding the collaboration of agile teams with their organizational environment. We will present and explain the identified concepts in this section.

In SAFe, cross-functional agile teams are called **technical teams** – they have all the necessary skills to define, build, test, and deliver products. We will discuss how these technical teams are supported by their environment, explain the differences in the concepts of system teams, shared services, and business teams, and discuss how they collaborate with the technical team to generate value.

The **Agile Release Train (ART)** is the mechanism used to synchronize different agile teams when it comes to joint development of a product increment. Different teams work on parts of the solution and have joint planning, daily standups, reviews, and retrospectives. **Solution trains** coordinate multiple ARTs to build complex solutions. Solution trains include organizational functions in the form of business teams.

Both system teams and shared services work in an ART to support value generation. **System teams** provide support in building and maintaining the proper environment for development, testing, or integration. System teams are usually not cross-functional teams, since they only focus on one aspect, e.g., taking over end-to-end testing. A system team can be dedicated to supporting one ART, or it could support all ARTs in a solution train.

**Shared services** are specialist roles that are needed in an ART, but that cannot be dedicated to a team full-time. Examples are data security experts or database administrators. Shared services may be responsible for supporting a certain ART, or even multiple ARTs across the enterprise. One way of working with the ART is that shared service staff join a team for a short period of time, which also has the advantage that knowledge is shared, so dependencies on the shared service team might be reduced in the future. Shared services occasionally form a separate team. Anyway, they join in all synchronization events of an ART and help resolve all issues related to backlog items where their experience is needed.

Thus, the difference is that system teams are incorporated as a team into the ART, while shared services are not dedicated to a specific team, but rather flexibly support an ART by providing their experience when and where it is needed.

**Business teams** provide support regarding infrastructure, contracting, supplier management, legal guidance, marketing, security, compliance topics, etc. They collaborate with technical teams and are aligned via shared objectives and the same cadence.

SAFe defines a **three-step process** for aligning business teams with technical teams. Business teams have to first adopt the agile mindset by applying the principles of the Agile Manifesto to their work. This allows for a shared value system in the whole company and an increased understanding on how technical teams work. Teams also apply the typical Scrum practices, like sprint planning, daily scrum, demo, and retrospective, and use the Scrum roles (Scrum Master and Product Owner). If the business teams have understood and live the mindset, they join the value stream. This means the way they work has to change; e.g., business teams have to collaborate more closely with the technical teams. This could happen by including a whole business team into an ART, having a business team work in a separate ART within a solution train, or by including single experts into an agile team. Finally, business teams need to identify their own agile way of working by defining specialized principles and practices. The old processes that conflict with the nature of agile need to be evolved to allow for better integration of the way of working of agile teams and the ART.

SAFe also mentions **other ways to handle dependencies** between an agile team and its environment. One specific interface appears between the development team and **operations**. DevOps is mentioned as the agile product delivery competency that is used in ART and solution trains. However, the operation side is not further explained. Another specific interface mentioned for solution trains is the one towards **suppliers**. Suppliers are part of the solution train, so they are required to work in an agile way, sharing the same cadence and participating in all events of the ART. They are treated like an individual ART that develops a subsystem or capabilities for the value stream. Another important dependency is towards **customers** that are involved at every level, e.g., with the help of the role of the Product Owner.

## Discussion and Related Work

In this section, the identified concepts for the collaboration between an agile team and its environment will be discussed and contrasted with related work. We will first discuss whether the existing guidance provided by the proposed concepts from SAFe is sufficient, especially highlighting any lack of guidance. Then we will discuss how SAFe provides guidance for the stepwise improvement of collaboration at the interface between technical and business teams aimed at agile collaboration. Finally, we will summarize the need for further research on collaboration between agile teams and the environment on which they depend.

### Guidance Regarding Collaboration.

In SAFe, the agile development team’s autonomy depends on support by shared services, support by system teams, and collaboration with business teams. SAFe defines these concepts with different levels of involvement in the development processes of an agile team. Some constraints are defined, such as working in the same cadence, or that synchronization happens throughout the events of the agile release train, such as joint planning, daily synchronization, product demonstration, and retrospectives. Thus, SAFe provides concrete guidance on the synchronization mechanisms that can be used.

However, it remains unclear whether the information and collaboration needs of all related stakeholders can be fulfilled by participating in these joint events. Some stakeholders might not benefit from participating in every event. On the other hand, satisfying the needs of all additional stakeholders might extend the scope and the intention of the normal sprint events, leading to increased time effort to conduct these meetings. Since product development happens in a cadence, it might not always be possible for shared service staff or business teams to attend multiple sprint events of several teams within the same ART.

There is also no concrete guidance on synchronization between a cross-functional agile delivery team and a functional system team, e.g., on how an agile software development team coordinates testing with one or multiple system teams that are responsible for end-to-end testing.

When setting up an ART, there is no help regarding the decision on what expertise has to be incorporated into the agile teams, or generally into the ART, and what expertise only collaborates with the agile team, e.g., in the form of business teams or shared services. This was also identified as a challenge in [11]. Developing criteria on when to use one or the other concept could be beneficial to practitioners. DevOps could be an example of how to bring two different functional teams closer together, and similar concepts could be developed for the collaboration between the agile team and other organizational functions.

Guidance is also needed on how agile teams should coordinate activities such as architecture or testing within a scaled product development. As an example, [12] investigated the collaboration between architects and agile teams - similar guidance is needed for other aspects.

SAFe only prescribes how to synchronize in the process, but does not talk about how to manage the information or products that are shared at the interface between an agile team and another party. Explicitly defining such so-called boundary objects that are exchanged across team borders [13] could improve collaboration, as SAFe does not provide information on what artifacts need to be exchanged or on which concrete information is shared.

### Transition to Agile Collaboration.

SAFe provides an example of how business teams align with the agile teams they support within an ART. A three-step process for integrating business functions into the ART is defined (cf. Sect. 3, business teams). First, business teams are required to understand agile and live the mindset themselves. In practice, companies that start with an agile transformation using SAFe adapt the framework to their own purpose and situation. Often, the surrounding organization is not touched and business functions continue to work in their established processes. A survey of SAFe adoptions also reported challenges regarding mindset change [14]. Guidance for assessing and improving the agility of teams is provided by [15].

As a second step, agile business teams need to join the ART in order to have a shared cadence and synchronization points. There is not much guidance on the way business functions have to collaborate with agile teams, or whether this collaboration is feasible for every business function.

Finally, business teams are supposed to improve their process in order to create their own agile way of working that harmonizes with the development flow of the ART. Guidance is missing for different business functions on what this collaboration could look like in detail.

### Summarizing the Need for Further Research.

In summary, the autonomy of an agile team highly depends on its environment. [16] also claims that the organizational culture and structures need to be adapted in order to increase the autonomy of an agile team. When synchronizing agile teams with their environment, the production and control structures influence the autonomy of agile teams [17]. Thus, it is important to be aware of the environment of an agile team, explicitly considering how to synchronize and what information to share.

Hence, it must first be understood with what parties an agile team has to collaborate. SAFe already mentions some dependent functions and responsibilities, but does not try to provide a complete view. More interfaces not explicitly addressed by SAFe exist, e.g., synchronizing agile development with a product line approach [18]. An initial overview of the dependencies of agile teams on their environment is provided by [2]. A possible next step for research would be to refine this classification of interfaces to understand what concrete dependencies exist in order to be able to find solutions.

SAFe provides synchronization concepts that could be used to improve the collaboration between an agile team and its environment, but there needs to be further research on when a certain concept is suitable for solving a certain dependency. In addition, SAFe does not provide guidelines about the content of the synchronization. Providing guidelines on what information to exchange or which artifacts to share at a certain interface could benefit practitioners. A final step would be to combine the “what” and the “how” to explain how the collaboration mechanisms can be used to convey certain information or artifacts.

## Conclusion and Future Work

Autonomous agile teams often have dependencies on their organizational environment. In order to improve the efficiency of these agile teams, collaboration with their environment has to be improved. The Scaled Agile Framework (SAFe) was reviewed to analyze what guidance exists for managing the dependencies of agile teams. SAFe provides concepts like system teams, business teams, or shared services that define different levels of involvement, but does not provide details on what efficient collaboration should look like.

In future work, we want to list existing dependencies by analyzing all scaling frameworks. It would also be interesting to look at other sources, such as PMBOK or CMMI, and evaluate how their recommendations are applicable to handling dependencies. Based on the results, those dependencies that need most research can be prioritized in order to find solutions for specific interfaces or identify strategies or patterns for collaboration that are commonly applicable.

## References

[1] VersionOne: 12th Annual State of Agile TM Report (2018) https://www.versionone.com/

[2] Theobald S, Diebold P, Garbajosa J, Wang X, Aguiar A (2018). Interface problems of agile in a non-agile environment. Agile Processes in Software Engineering and Extreme Programming.

[3] Marner K, Theobald S, Wagner S, Przybyłek A, Morales-Trujillo ME (2020). Release planning in a hybrid project environment. Advances in Agile and User-Centred Software Engineering.

[4] Krieg, A., Theobald, S., Küpper, S.: Erfolgreiche agile Projekte benötigen ein agiles Umfeld. In: Mikuzs, M., Volland, A., Engstler, M., Hanser, E., Linssen, O. (eds.) Projektmanagement und Vorgehensmodelle 2018 - Der Einfluss der Digitalisierung auf Projektmanagementmethoden und Entwicklungsprozesse, pp. 217–222. Gesellschaft für Informatik, Bonn (2018)

[5] Moe NB, Stray V, Hoda R, Hoda R (2019). Trends and updated research agenda for autonomous agile teams: a summary of the second international workshop at XP2019. Agile Processes in Software Engineering and Extreme Programming – Workshops.

[6] Stray, V., Moe, N.B., Hoda, R.: Autonomous agile teams: challenges and future directions for research. In: Proceedings of the 19th International Conference on Agile Software Development: Companion, pp. 1–5. ACM, Porto (2018)

[7] Bass JM, Hoda R (2019). Future trends in agile at scale: a summary of the 7^th^ international workshop on large-scale agile development. Agile Processes in Software Engineering and Extreme Programming – Workshops.

[8] Barroca L, Dingsøyr T, Mikalsen M, Hoda R (2019). Agile transformation: a summary and research agenda from the first international workshop. Agile Processes in Software Engineering and Extreme Programming – Workshops.

[9] Diebold, P., Schmitt, A., Theobald, S.: Scaling agile: how to select the most appropriate framework. In: Proceedings of the 19th International Conference on Agile Software Development: Companion (XP 2018), pp. 1–4. Association for Computing Machinery, New York (2018). Article 7. 10.1145/3234152.3234177

[10] Scaled Agile Framework (2019). http://www.scaledagileframework.com/. Accessed 01 Dec 2019

[11] Putta A, Paasivaara M, Lassenius C, Kruchten P, Fraser S, Coallier F (2019). How are agile release trains formed in practice? A case study in a large financial corporation. Agile Processes in Software Engineering and Extreme Programming.

[12] Uludağ Ö, Kleehaus M, Erçelik S, Matthes F, Kruchten P, Fraser S, Coallier F (2019). Using social network analysis to investigate the collaboration between architects and agile teams: a case study of a large-scale agile development program in a german consumer electronics company. Agile Processes in Software Engineering and Extreme Programming.

[13] Wohlrab, R., Pelliccione, P., Knauss, E., Larsson, M.: Boundary objects in Agile practices: continuous management of systems engineering artifacts in the automotive domain. In: Proceedings of the 2018 International Conference on Software and System Process (ICSSP 2018), pp. 31–40. Association for Computing Machinery, New York (2018). 10.1145/3202710.3203155

[14] Laanti M, Kettunen P, Hoda R (2019). SAFe adoptions in Finland: a survey research. Agile Processes in Software Engineering and Extreme Programming – Workshops.

[15] Guckenbiehl, P., Theobald, S.: Assessment of agile culture. In: PVM 2019, p. 165 (2019)

[16] Spiegler SV, Heinecke C, Wagner S, Hoda R (2019). The influence of culture and structure on autonomous teams in established companies. Agile Processes in Software Engineering and Extreme Programming – Workshops.

[17] Mikalsen M, Næsje M, Reime EA, Solem A, Hoda R (2019). Agile autonomous teams in complex organizations. Agile Processes in Software Engineering and Extreme Programming – Workshops.

[18] Hohl P, Theobald S, Becker M, Stupperich M, Münch J, Kuhrmann M (2018). Mapping agility to automotive software product line concerns. Product-Focused Software Process Improvement.

